# Research on the Absorption Performance of Glass Fiber Fabric Composites Coated with Nickel by Magnetron Sputtering

**DOI:** 10.3390/polym18161979

**Published:** 2026-08-14

**Authors:** Zhuohui Zhou, Yanli Wang, Mengyu Zhou, Zhiyong Wang, Yan Zhao

**Affiliations:** 1 School of Materials Science and Engineering, Beihang Universtiy, Beijing 102206, China; zhouzhuohui@126.com; 2 Beijing Institute of Aeronautical Materials, Beijing 100095, China; 3 Jiangxi Hongdu Aviation Industry Group Co., Ltd., Nanchang 330024, China

**Keywords:** microwave absorption performance, magnetron sputtering, glass fiber fabric composites, nickel coating

## Abstract

This study focuses on the deposition of nickel thin-films onto glass fiber fabric via DC magnetron sputtering and explores their potential for broadband microwave absorption applications. A total of twelve laminate samples were prepared by integrating the coated fabrics with epoxy resin, with sputtering powers ranging from 0.5 to 2 kW and deposition times ranging from 10 to 90 min. The microstructure, surface resistance, electromagnetic parameters, and microwave absorption performance were systematically characterized using SEM, XRD, four-point probe measurements, and vector network analysis, supplemented by the Lorentz model fitting and simulation validation. The results indicate that the nickel coatings exhibit a non-uniform arc-like morphology, with preferential growth along the (111) crystallographic plane, while the (200) and (220) planes form under specific conditions. The surface resistance reaches up to 10^8^ Ω·m, suggesting the absence of a continuous conductive network. Electromagnetic parameter analysis reveals that the laminates display dielectric-loss-dominated microwave absorption, and the Lorentz fitting identifies double resonance peaks under prolonged or high-power sputtering. The addition of a dielectric matching layer further enhances the absorption performance. All samples achieve wideband absorption within the Ku-band. Notably, the samples prepared at 1 kW for 30 min and at 1 kW for 90 min both exhibit a reflectivity of ≤−10 dB across the entire 8–18 GHz frequency range. The experimental results are in good agreement with simulations. The bulk density of the laminates is approximately 1.8 g/cm^3^. These findings confirm that magnetron-sputtered nickel-coated continuous glass fiber fabrics hold considerable promise for wideband microwave absorption applications.

## 1. Introduction

With the development of communication technology, especially the wide application of 5G technology, communication frequency bands have evolved from MHz to GHz. The problem of electromagnetic pollution in daily life has become increasingly serious, not only causing mutual interference among electronic devices but also having certain impacts on human health [1]. Microwave absorbing materials are able to convert electromagnetic wave energy into thermal energy. They are mainly classified into coating absorbing materials and structural absorbing materials based on their application forms [2,3]. Coating absorbing materials [4,5,6], due to their ease of construction, have developed rapidly initially. However, their poor performance and limitations on coating thickness have increasingly restricted their development. Structural absorbing materials, on the other hand, refer to materials that possess both electromagnetic wave absorption capabilities and certain mechanical load-bearing capacity, with continuous fiber-reinforced resin composites being representative. Since continuous fiber-reinforced resin composites are composed of resin matrix and reinforcing fibers, there are two ways to endow structural composites with absorbing functions: the first is to add absorbents to the resin matrix to give it certain electromagnetic wave absorption capacity; the second is to enhance the absorbing performance of reinforcing fibers. Among these approaches, incorporating absorbents into the resin matrix represents one of the earliest investigated technical strategies. Specifically, dielectric-loss-type absorbents [7,8,9,10] such as carbon black, carbon nanotubes, and graphene can be embedded within the matrix, while magnetic loss-type absorbents [11,12,13,14]—including ferrites, carbonyl iron, and permalloy—are also commonly introduced to enhance electromagnetic wave absorption performance.

Over more than a decade of development, incorporating a high proportion of absorbent fillers—such as dielectric-loss carbonaceous materials or magnetic loss ferrites and carbonyl iron—into a polymer resin matrix does indeed enable broadband microwave absorption in composites [15,16]. However, this technical route has always faced inherent trade-offs. On one hand, high filler loadings, though beneficial for loss performance, inevitably increase composite density and undermine lightweighting advantages [17,18]; meanwhile, excessive particles disrupt the resin cross-linked network, leading to notable degradation in flexural strength and interlaminar shear properties. On the other hand, reducing the filler content rapidly deteriorates impedance matching, causing a sharp decline in absorption peak intensity and effective bandwidth—a classic “trade-off” dilemma [19]. More critically, issues such as particle agglomeration, unstable interfacial polarization, and mismatched thermal expansion between fillers and resin are often oversimplified in laboratory studies, resulting in significant discrepancies between lab-scale performance and actual engineering batches [20]. The second technical route-depositing absorbent coatings (e.g., dielectric coatings like carbon nanotubes and graphene, or magnetic coatings like ferrites and magnetic alloys) onto reinforcing fiber surfaces aims to circumvent the mechanical property losses associated with matrix modification. This approach effectively reduces overall density, and in principle, thin-film magnetic metals can surpass the Snoek limit, enhancing low-frequency permeability [21]. Nevertheless, existing deposition techniques (electroplating, electroless plating, sol–gel, etc.) often struggle to precisely control coating uniformity and thickness, leading to inhomogeneous distributions within fiber bundles; localized overly conductive networks may worsen impedance mismatch instead. Moreover, most studies rely on a single loss mechanism, lacking synergistic dielectric—magnetic design, whereas hybrid coating processes are overly complex and costly, and the long-term reliability of coating-fiber interfacial adhesion remains inadequately verified-severely hampering translation from laboratory to industrial application [22].

In sharp contrast, our proposed thin-film structured magnetic metal-deposited continuous fiber fabric achieves a systematic leap in absorbing performance while retaining the inherent mechanical advantages of fiber-reinforced composites. The significant advancements are manifested in the following aspects. First, through optimized deposition parameters, a uniform, dense magnetic metal (e.g., nickel, cobalt, or their alloys) thin-film with thickness tunable from nanometers to micrometers can be formed on each individual continuous fiber surface [23]. This structure not only avoids particle agglomeration and interfacial defects but also endows the material with pronounced shape anisotropy, allowing permeability to far exceed the Snoek limit in the GHz range-effectively compensating for the weak low-frequency response of conventional particulate fillers [24]. Second, the conductive and magnetic thin-film simultaneously provides dielectric loss (from the conductive network) and magnetic loss (dominated by natural resonance and eddy-current loss). By adjusting film thickness and fabric-weaving architecture, electromagnetic parameters can be flexibly tuned to achieve excellent impedance matching and broadband absorption, far outperforming single-loss-mechanism coatings or filler-filled composites. Third, because the absorbing function is integrated into the fibers themselves rather than the resin matrix, the matrix retains its original low density, high toughness, and good processability [25]. As a result, the final composite no longer forces a compromise between absorption performance and mechanical load-bearing capacity—both are attained simultaneously. Fourth, this scheme uses continuous fiber fabric as the substrate, and the deposition process is highly compatible with existing textile preform manufacturing routes, facilitating scalable production and co-curing with complex shapes, thus significantly lowering the barrier to engineering translation [26]. In summary, our approach not only fundamentally overcomes the inherent bottlenecks of traditional filler-based and fiber-coating methods—such as inhomogeneity, poor mechanical designability, and mismatched impedance—but also provides a practical and new pathway toward low-frequency, broadband, lightweight, and high-load-bearing structural absorbing materials [27].

The magnetron sputtering coating process is currently one of the most widely used techniques for precision coating in industrial applications. It possesses several critical advantages. First, the range of raw materials suitable for fabricating magnetron sputtering cathode targets is extensive, covering nearly all metals, alloys, and ceramic materials [28,29]. This broad material compatibility provides a robust foundation for selecting appropriate absorbers. Second, the process achieves high deposition rates while producing dense films with excellent adhesion to the substrate. These characteristics facilitate large-scale, high-efficiency industrial manufacturing. Moreover, the strong interfacial adhesion between the film and the substrate plays a crucial role in preserving the mechanical integrity of the resulting absorbing composite materials. Nam, Y.-W. et al. [30] developed a new radar-absorbing structure by applying a silver-sputtering coating on glass fabric. A thin and lightweight microwave-absorbing structure was designed and fabricated using silver-coated glass fabric with thicknesses of 1.82, 1.84, and 1.95 mm, showing excellent performance in the X-band (8.2~12.4 GHz), without incorporating conductive carbon nanomaterials into the resin matrix. Wang, X. et al. [31] proposed a microwave-absorbing structure by nichrome coated quartz fiber fabric (NFF) and pristine quartz fiber fabric (PFF). The so-made microwave-absorbing structure is thin and lightweight, and exhibits good absorption performance in X and Ku frequency bands, with wide bandwidth below −10 dB from 8.5 to 15.5 GHz, and reaches the lowest reflectivity of −53 dB at 11.48 GHz. Zou, Y. et al. [32] fabricated the hierarchical structures of carbonized melamine foam modified by FeNi alloy and silicon layer (CMF/FeNi-SiO2) by a magnetron sputtering method. The minimum reflection loss of CMF/FeNi-SiO2 with an absorption thickness of 4.1 mm was about −53 dB at 3.38 GHz and reached an effective absorption bandwidth of 0.93 GHz (from 2.93 GHz to 3.86 GHz).

Nickel (Ni), as an important magnetic metallic material, exhibits significant application advantages in the field of microwave absorption [33]. On one hand, nickel possesses both excellent magnetic loss and dielectric-loss capabilities, enabling a synergistic dielectric/magnetic loss system that effectively optimizes impedance matching and enhances electromagnetic wave attenuation. On the other hand, nickel has a high Snoek limit, allowing it to maintain superior magnetic response in the microwave frequency range, especially in the GHz band [34]. Furthermore, nickel is abundant in resources, mature in preparation processes, and has good corrosion resistance and mechanical properties, which facilitate low-cost fabrication and practical applications of absorbing materials [35]. In recent years, researchers have significantly improved the microwave absorption performance of nickel-based materials through microstructure optimization, alloying, and composite design with other functional materials such as carbon fibers, graphene, and metal oxides [35,36,37].

Based on these advantages, this study employs magnetron sputtering to deposit nickel nano-films on the surface of continuous reinforcing fiber fabrics. By adjusting the sputtering power and deposition time, nickel-coated fiber fabric samples with varying process parameters are fabricated and subsequently laminated with epoxy resin. The microstructure of the Ni-coating, crystal growth behavior, electromagnetic parameters of the laminate, and its microwave absorption performance were systematically investigated. Notably, this study introduces a novel sputtering-based strategy that overcomes the key limitations of conventional electroless plating—such as non-uniform coverage and poor process controllability by enabling precise tailoring of coating morphology and electrical properties through simple parameter adjustment. In addition, for the first time, we apply a phenomenological model to fit the measured electromagnetic parameters (complex permittivity and permeability), which enables a reliable extraction of intrinsic loss contributions and frequency-dependent behavior, offering a robust predictive tool that goes beyond empirical fitting. Furthermore, an optimization design methodology for microwave absorption performance of absorbing composite materials utilizing nano-film/enhanced fiber as the absorbing medium was explored, which uniquely combines experimental data with theoretical modeling to achieve both lightweight and broadband absorption-a critical challenge that remains unsolved by most existing metal—fabric composites. This work establishes a theoretical foundation for the practical application of lightweight, broadband absorbing composite materials and offers a scalable, cost-effective pathway.

## 2. Materials and Methods

### 2.1. Materials

The primary raw materials used in this study include EW100A-100a grade glass fiber fabric with a plain weave structure. Both the warp and weft fiber densities are 20 threads per centimeter. The glass fiber fabric was supplied by the Nanjing Glass Fiber Research Institute (Nanjing, China). A nickel target with a purity of 99.99% and dimensions of 1000 mm in length, 100 mm in width, and 2 mm in thickness, bonded to a 1 mm thick copper backing plate, was provided by Zhongnuo New Materials Co., Ltd. (Beijing, China). The resin employed is a medium-temperature epoxy resin (grade ME-301) with a density of 1.2 g/cm^3^, sourced from Zhengzhou University (Zhengzhou, China). Additional auxiliary materials, such as acetone and deionized water, were used for cleaning purposes.

### 2.2. Experimental Process

#### 2.2.1. Preparation of Fiber Glass Fabric

The glass fiber fabric was first cut into pieces of 1000 mm × 600 mm. These pieces were then ultrasonically cleaned in a 1:1 mixture of acetone and deionized water for 30 min, with occasional gentle stirring using a glass rod to remove organic solvents, dust, and other surface impurities. After cleaning, the fabric was rinsed three times with deionized water, dried in an oven at 50 °C for 15 min, and finally stored in sealed sample bags placed in a desiccator for later use.

#### 2.2.2. Preparation of Magnetron Sputtering

**Figure 1:**
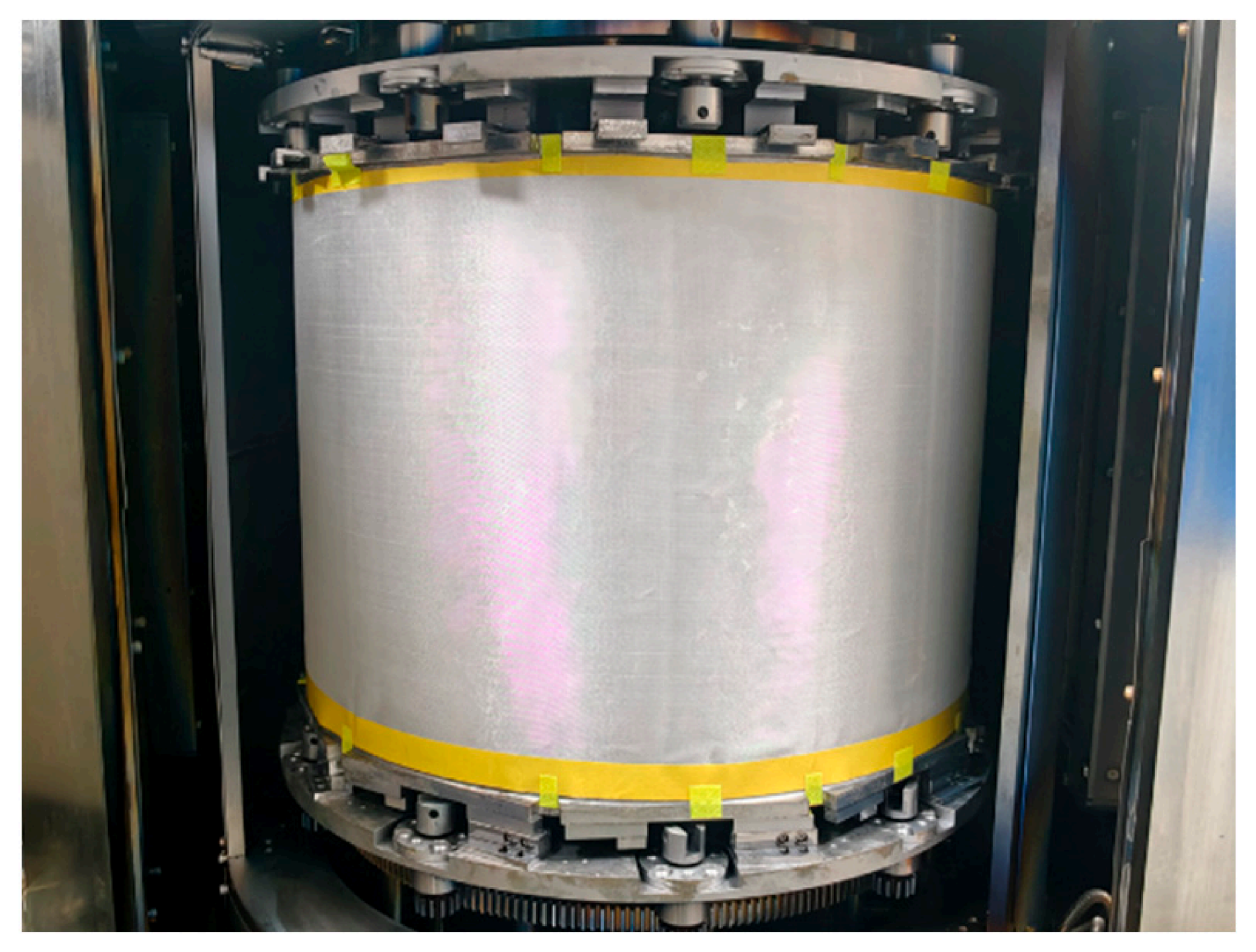
The glass fiber fabric mounted on the substrate holder.

Nickel coatings were deposited using a DC magnetron sputtering system (manufactured by Beijing Shiliyuan Technology Development Co., Ltd. (Beijing, China), model SP-3815ASI). The glass fiber fabric was mounted on a cylindrical substrate holder and secured with tape (see Figure 1), with a target-to-substrate distance of 100 mm. The deposition was carried out under a base vacuum of 1 × 10^−3^ Pa, an argon flow rate of 60 SCCM, a substrate rotation speed of 2 rpm, and a working pressure of 0.5 Pa. By varying the sputtering power (0.5–2 kW) and deposition time (10–90 min) while keeping these base parameters constant, a series of Ni-coated fabric samples was obtained. The detailed process parameters for each sample are listed in Table 1.

#### 2.2.3. Preparation of Prepreg Materials

Prepreg is a ready-to-mold fiber-reinforced material that contains a partially cured resin, requiring only heat and pressure to complete the curing process. The epoxy resin was first mixed with acetone in a 1:1 ratio. The resulting solution was then sprayed evenly onto both surfaces of the Ni-coated glass fiber fabric using a spray gun equipped with a 0.5 mm nozzle at a pressure of approximately 20 kPa. Each pass applied about 10–15 g/m^2^ of resin. After each layer dried, additional coats were applied until the overall surface density of the prepreg increased by 70 g/m^2^. The prepared prepreg was then stored for subsequent lamination.

#### 2.2.4. Preparation of Laminates

The prepreg layers were stacked in a [0]_20_ ply sequence, vacuum-bagged, and cured in an autoclave. The curing cycle started with applying a vacuum to reach ≤−0.0095 MPa. At room temperature, the pressure was increased at 0.03 MPa/min to 0.3 MPa, while simultaneously the temperature was raised at 1.5 °C/min to 80 °C and held for 30 min. The temperature was then further increased at 1 °C/min to 135 °C and maintained for 120 min. Finally, the system was cooled down at 1 °C/min under maintained pressure until the temperature reached 60 °C, after which the cured laminate was removed from the autoclave.

#### 2.2.5. Preparation of Test Samples

**Figure 2:**
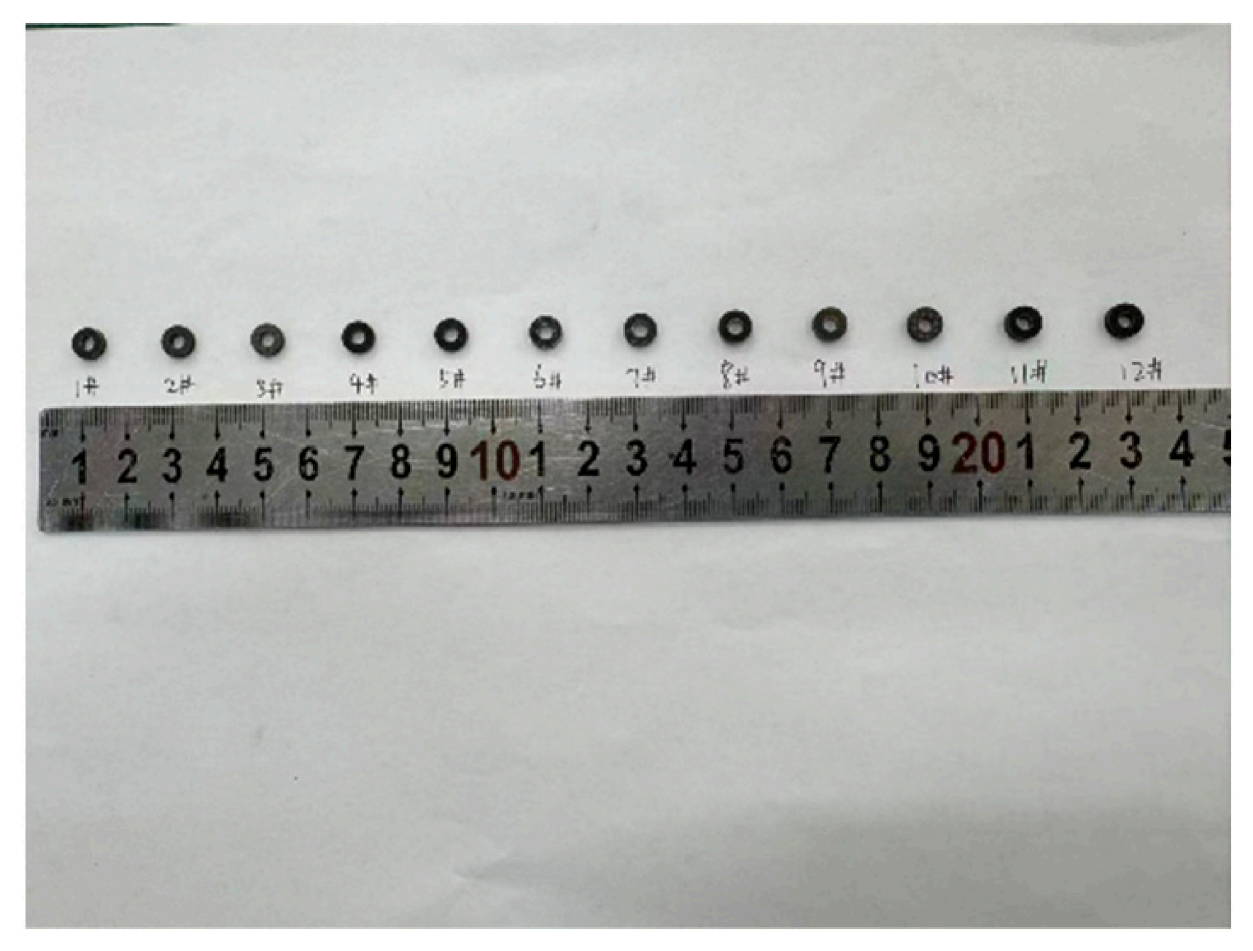
The electromagnetic parameter test samples and the handwritten 1#~12# represent different samples.

From the cured laminates, toroidal specimens with an outer diameter of 7 mm and an inner diameter of 3 mm were machined using a computer numerical control (CNC) machining center (see Figure 2). The electromagnetic parameters of these specimens were then measured using the coaxial transmission/reflection method in accordance with GB/T 5597.

#### 2.2.6. The Testing Equipment

**Figure 3:**
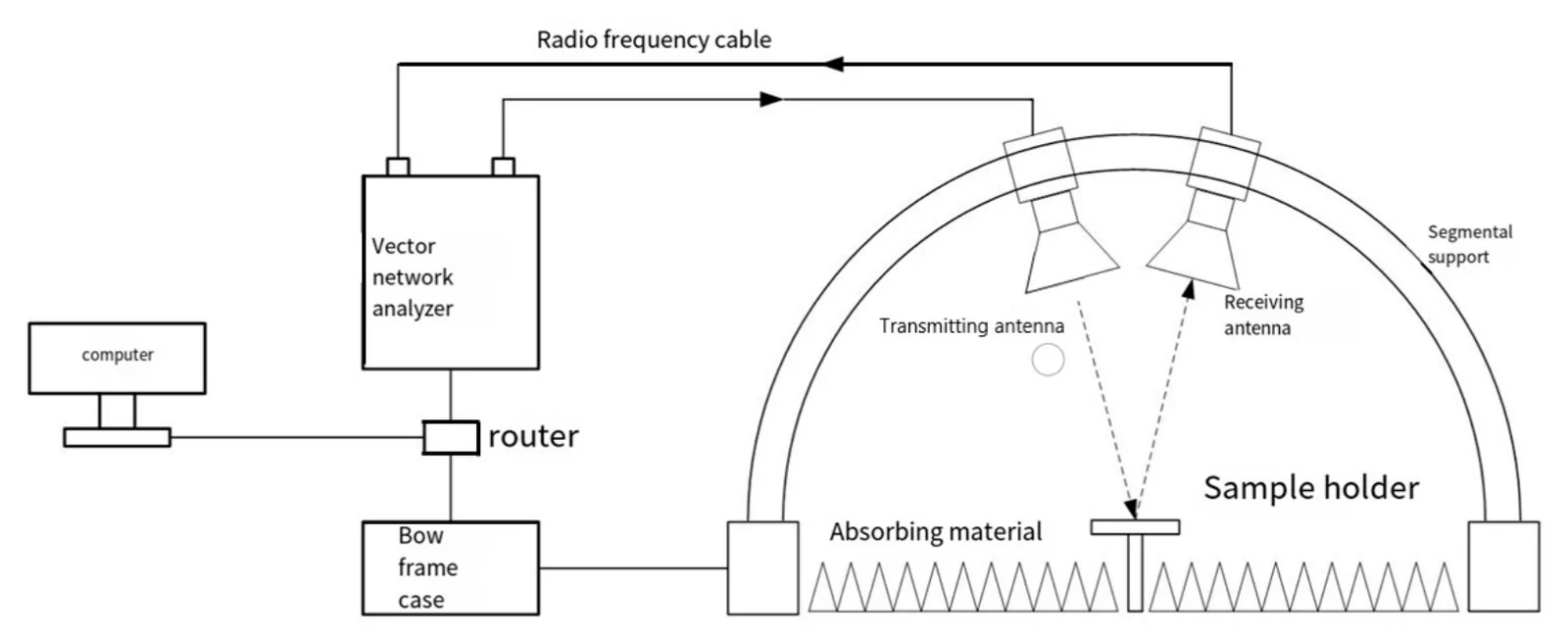
The Arch measurement system.

The testing equipment used in this paper is shown in Table 2:

The scanning electron microscope operated at a voltage of 15 kV with a working distance of approximately 7–8 mm, and the magnification was adjusted according to the morphological features. The X-ray diffractometer used a Cu target, operated at 45 kV, with a step size of 0.02°, and a scan rate of 20°/min. The vector network analysis was conducted over the frequency range of 1–18 GHz, while the reflectivity measurements using the Arch measurement system (Figure 3) were performed from 2 to 18 GHz, with a background level of −40 dB.

## 3. Results and Discussion

### 3.1. SEM Test Results of Ni-Coated Glass Fiber Fabric

**Figure 4:**
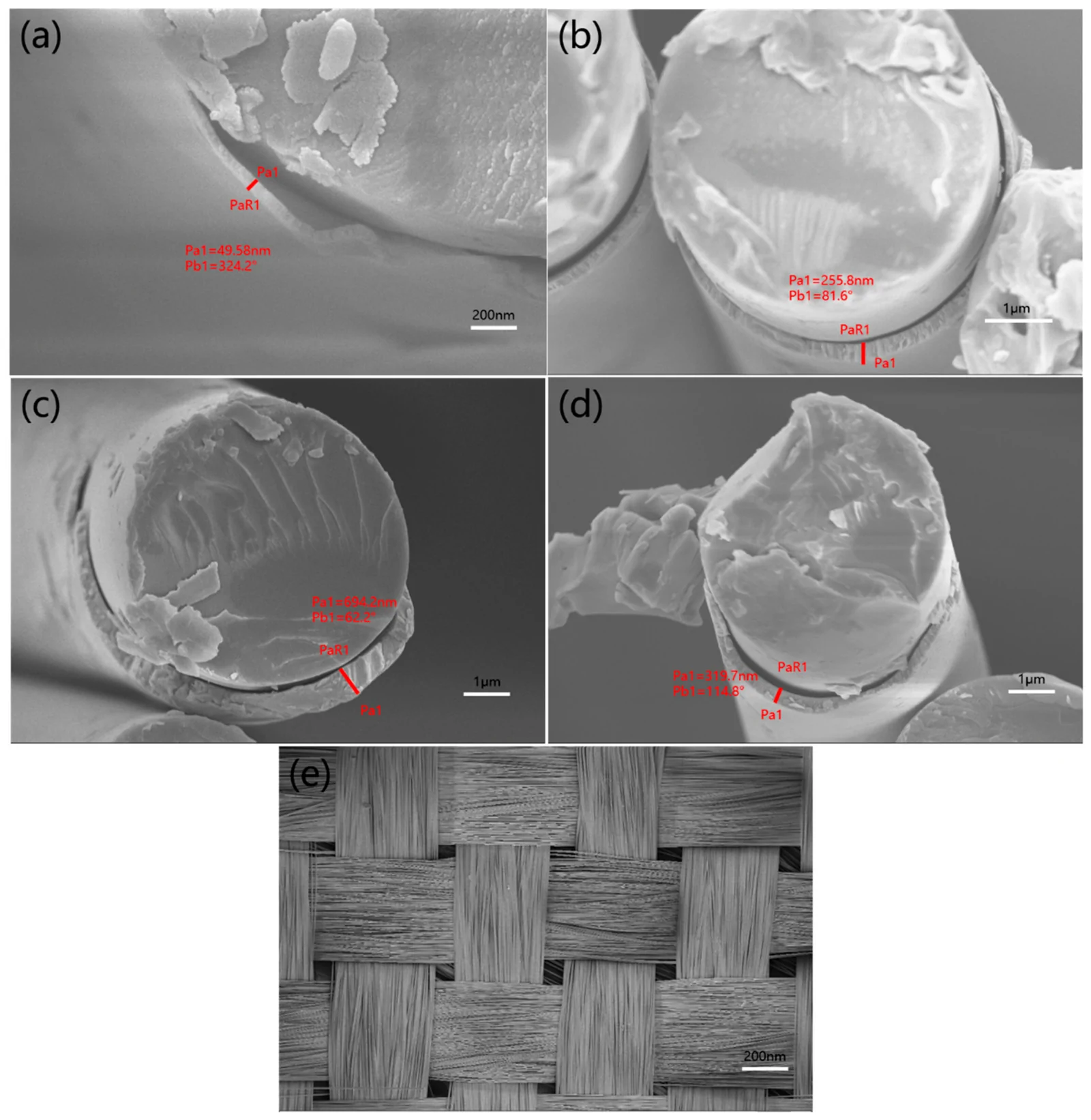
SEM test results of typical samples (**a**) 1#, (**b**) 6#, (**c**) 8#, (**d**), 10#, and (**e**) 12#.

Figure 4 presents the SEM results of the Ni-coated glass fiber fabric, where Figure 4a to Figure 4d correspond to typical Samples 1#, 6#, 8#, and 10#, respectively. Figure 4e presents the as-received plain weave architecture, featuring orthogonal interlacing of ±90° fiber tows. The SEM images reveal that the Ni-coating accumulates only on the side of the fiber fabric facing the Ni target. This results in a circular arc-shaped distribution with a higher center and lower edges, and the coated area does not exceed half of the surface area of a single fiber. As both sputtering time and power increase, the overall coating thickness exhibits an increasing trend.

For each sample, we randomly selected five positions and measured the thickness of the coating. The test results are shown in Table 3.

As shown in the above table, the thickness of the fiber coating varies significantly across different positions within the same sample. This observation indicates that the Ni nano-coating does not deposit uniformly on the fibers. Only the fibers located on the surface layer of the fiber bundle and directly facing the target can achieve the maximum coating thickness. For the inner layer fibers, the surface layer fibers create a shielding effect, allowing only a limited number of atoms to pass through the inter-fiber gaps and deposit onto the inner fibers. Moreover, as both sputtering power and time increase, the variation in coating thickness within a single sample becomes more pronounced.

**Figure 5:**
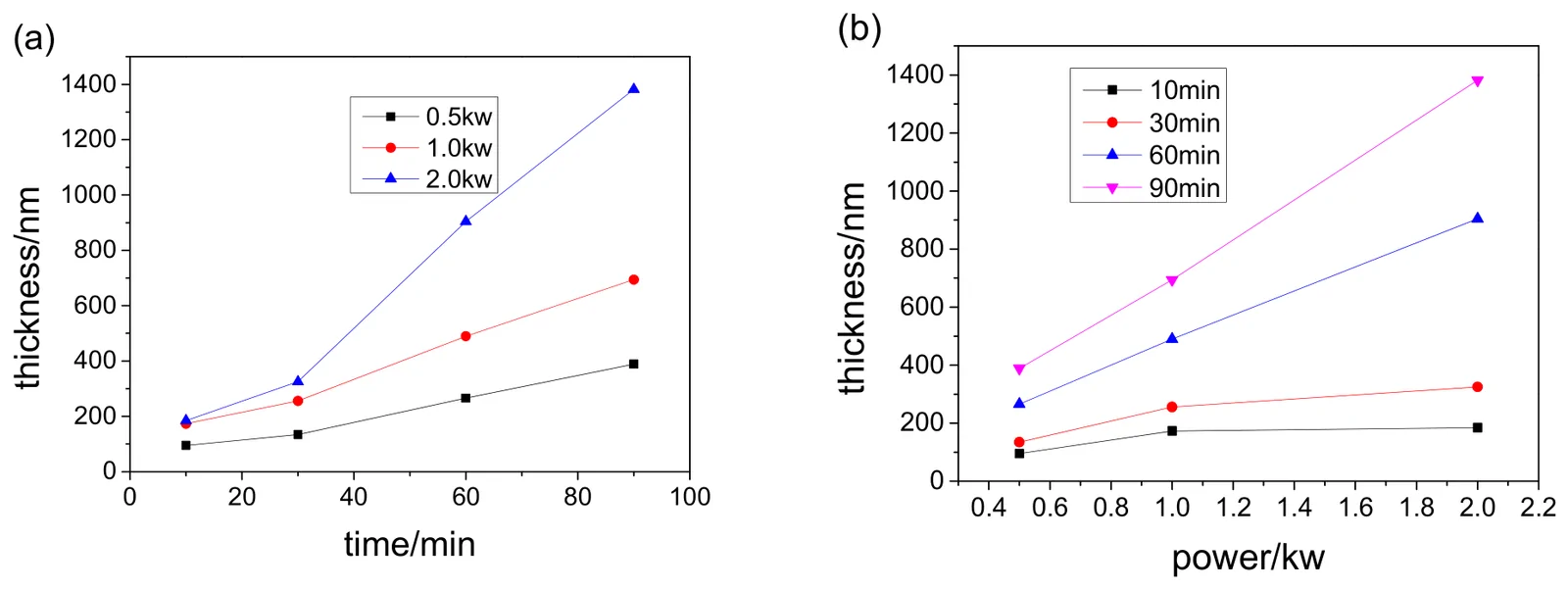
Influence of process parameters on the maximum thickness of the coating layer (**a**) sputtering time (**b**) sputtering power.

Figure 5 illustrates the variation trend of coating thickness with respect to sputtering power and sputtering time. When the sputtering power is held constant, at lower power levels, the coating thickness increases steadily as the sputtering time extends. At higher power levels, the growth rate of the coating thickness accelerates with increasing sputtering time. Conversely, when the sputtering time is fixed, within a relatively short duration, the growth rate decreases slightly as the sputtering power increases. However, over a longer sputtering time period, the coating thickness exhibits a stable growth trend, with a nearly constant growth rate as the sputtering power increases.

### 3.2. XRD Test Results of Ni-Coated Glass Fiber Fabric

**Figure 6:**
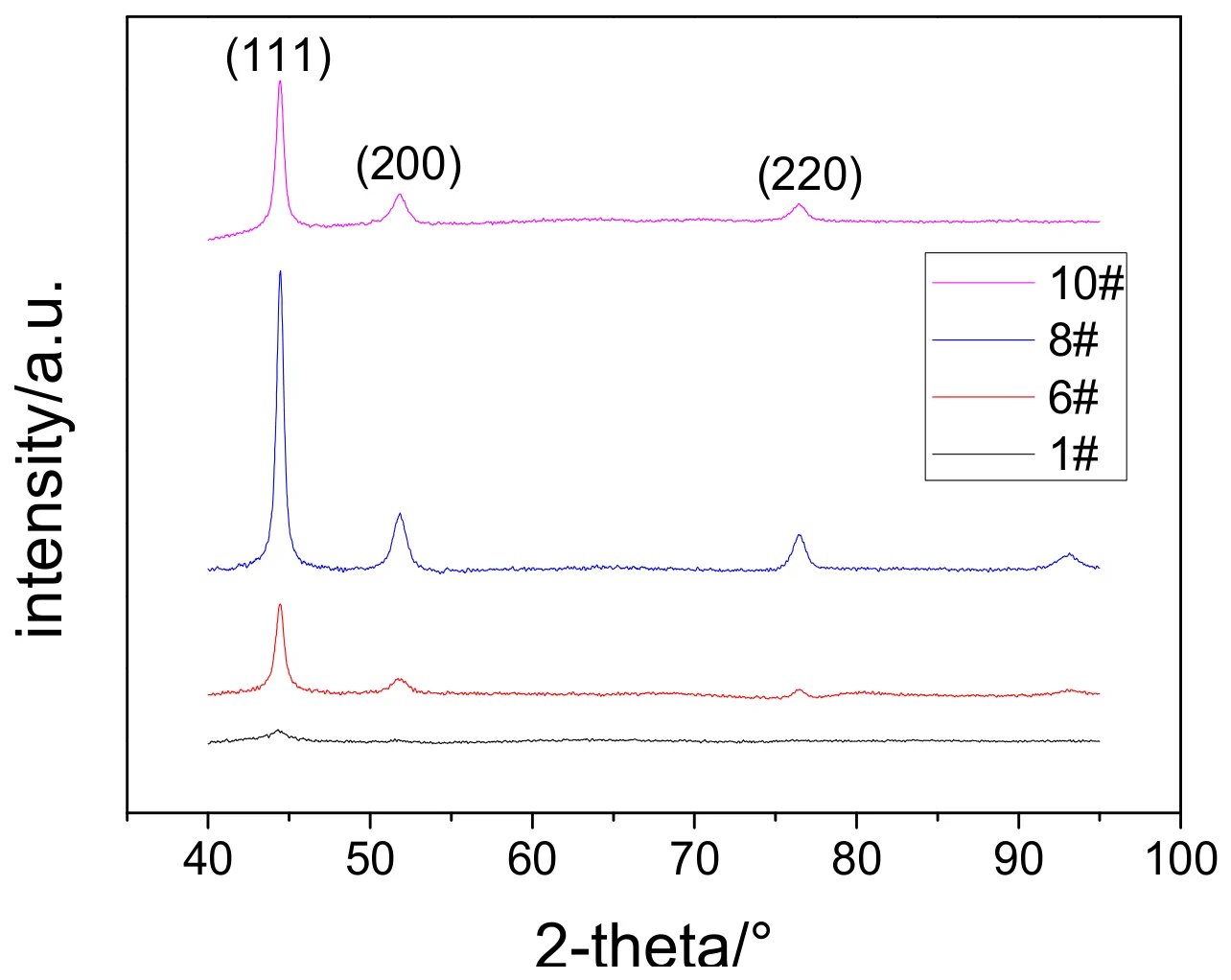
XRD results of typical samples.

Figure 6 presents the XRD patterns of the coating on the fiber fabric, which correspond to Samples 1#, 6#, 8#, and 10#, respectively. The diffraction peak angles and intensities for each sample are summarized in Table 4. The diffraction peaks are predominantly observed within the angular ranges of 44.2° to 44.5°, 51.7° to 52.0°, and 76.2° to 76.5°. Among these, the peak located between 44.2° and 44.5° exhibits the highest intensity. By comparing these peaks with the theoretical diffraction positions of pure nickel, it can be concluded that the peak between 44.2° and 44.5° corresponds to the (111) crystallographic plane of Ni, the peak between 51.7° and 52.0° corresponds to the (200) plane, and the peak between 76.2° and 76.5° corresponds to the (220) plane. The minor angular deviations are attributed to instrumental calibration errors.

The lattice parameter derived from the XRD results is approximately 3.52 Å, which is in good agreement with the reported value for face-centered cubic nickel (JCPDS PDF #04-0850; a = 3.5238 Å). This result is consistent with the presence of a nickel phase in the deposited coating. However, it should be noted that XRD analysis alone does not conclusively prove the absence of trace impurities or minor secondary phases.

### 3.3. Resistance Analysis of Ni-Coated Fiber Fabric

The surface resistance of the coated fabric was measured using the four-probe method, and the results are summarized in the following table. The data indicate that the surface resistance values are relatively high, on the order of 10^8^ Ω, classifying the material as an insulator, as shown in Table 5. Furthermore, no variation trend can be observed in the resistance under different process parameters. This may be attributed to the fabric structure, which is relatively loose, causing measurement fluctuations depending on the probe placement. Nevertheless, the high-resistance values suggest that no continuous conductive network has formed between the coatings, which is closely related to the fabric’s weaving structure. In plain weave fabric, warp and weft fibers alternate, creating intersections where the nano-coatings tend to be discontinuous during magnetron sputtering. As a result, a conductive path cannot be established, leading to the high-resistance behavior of the Ni-coated fabric.

### 3.4. Analysis of Electromagnetic Parameters of Laminated Plates

**Figure 7:**
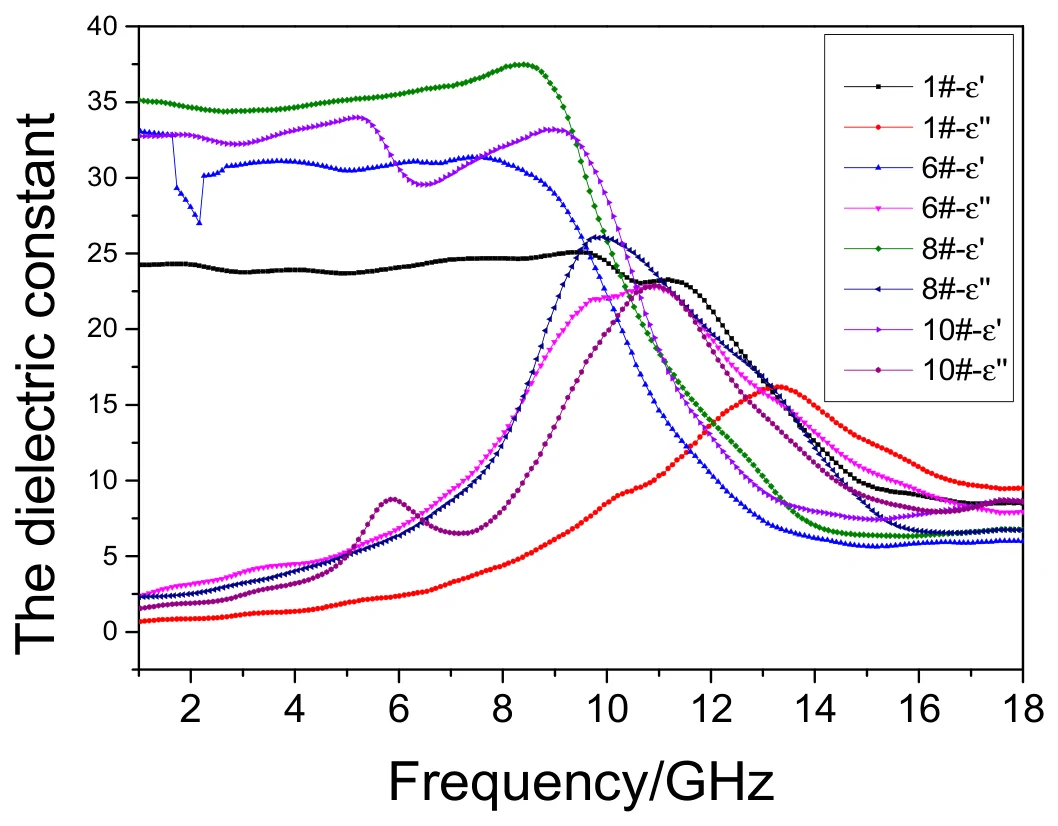
Electromagnetic parameters of typical samples.

Figure 7 presents the electromagnetic parameters of the laminated plates. The data indicate that the Ni-coated laminated plates exhibit characteristics of electrically lossy materials. Variations in sputtering time and sputtering power do not result in significant changes in the electromagnetic parameters, particularly the magnetic permeability. Across all sputtering conditions, the real part of the magnetic permeability remains approximately between 1.1 and 1.3, suggesting weak magnetic properties. The real part of the dielectric constant shows slight fluctuations but is generally maintained within the range of 25 to 35. According to our analysis, this behavior can be attributed to the high surface resistance of the fiber fabric (see Table 5 for the measured values). This high resistance arises from the macroscopic discontinuity of the conductive network, which is inherently dictated by the orthogonal weave structure of the glass fibers: the coating forms on individual fiber strands but does not bridge across the woven intersections in a continuous manner. Once the individual fiber units are already coated, extending the sputtering time does not increase the length of the continuous conductive path on each fiber, but merely increases the thickness of the nickel deposit on the already-coated surfaces. As a result, the overall conductive connectivity remains essentially unchanged, and consequently, both the dielectric constant and the magnetic permeability exhibit negligible variation with increased deposition time.

The electromagnetic parameters reveal that the real part of the dielectric constant exhibits a significant decrease within the frequency range of 8 to 18 GHz, while the imaginary part reaches a maximum in the corresponding frequency region where the real part declines. This behavior may be consistent with the Lorentz resonance model, and the complex dielectric constant can be expressed as follows [38]:

$$\varepsilon^{*}\left(\omega \right)=\varepsilon_{\infty }+\frac{\Delta \varepsilon \omega_{0}^{2}}{\omega_{0}^{2}-\omega^{2}+j\gamma \omega }$$

Here, Δε represents the change in dielectric constant, $\omega_{0}$ is the resonant frequency, γ is the damping coefficient, and $\varepsilon_{\infty }$ is the high-frequency dielectric constant.

The real part of the dielectric constant is as follows:

$$\varepsilon^{\prime }\left(\omega \right)=\varepsilon_{\infty }+\frac{\Delta \varepsilon \omega_{0}^{2}\left(\omega_{0}^{2}-\omega^{2}\right)}{{\left(\omega_{0}^{2}-\omega^{2}\right)}^{2}+\gamma^{2}\omega^{2}}$$

The imaginary part of the dielectric constant is as follows:

$$\varepsilon^{\prime \prime }\left(\omega \right)=\frac{\Delta \varepsilon \omega_{0}^{2}\gamma \omega }{{\left(\omega_{0}^{2}-\omega^{2}\right)}^{2}+\gamma^{2}\omega^{2}}$$

When $\omega =\omega_{0}$, the real part $\varepsilon^{\prime }\left(\omega \right)$ undergoes a sharp change, and the frequency of the maximum loss peak of the imaginary part $\varepsilon^{\prime \prime }\left(\omega \right)$ can be written as follows:

$$\omega_{max}=\omega_{0}\sqrt{1-\frac{\gamma^{2}}{2\omega_{0}^{2}}}\;(\gamma <\sqrt{2}\omega_{0})$$

$$f_{max}=\omega_{max}/2\pi$$

**Figure 8:**
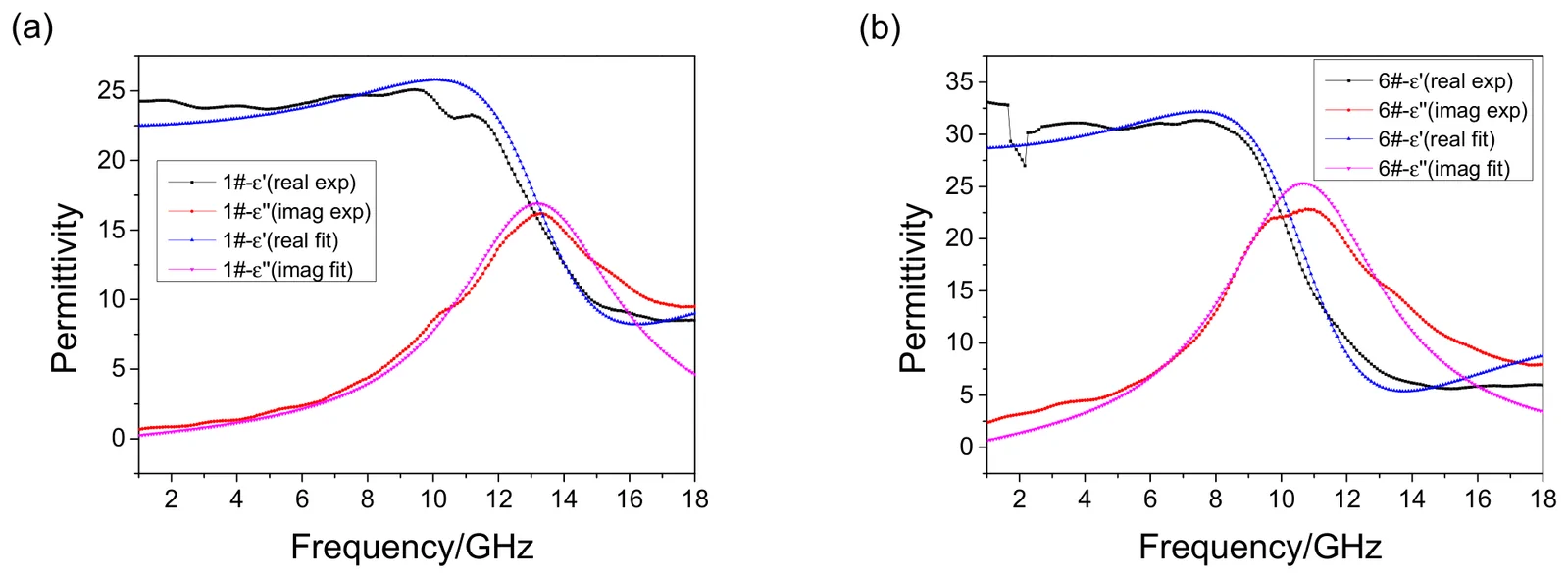

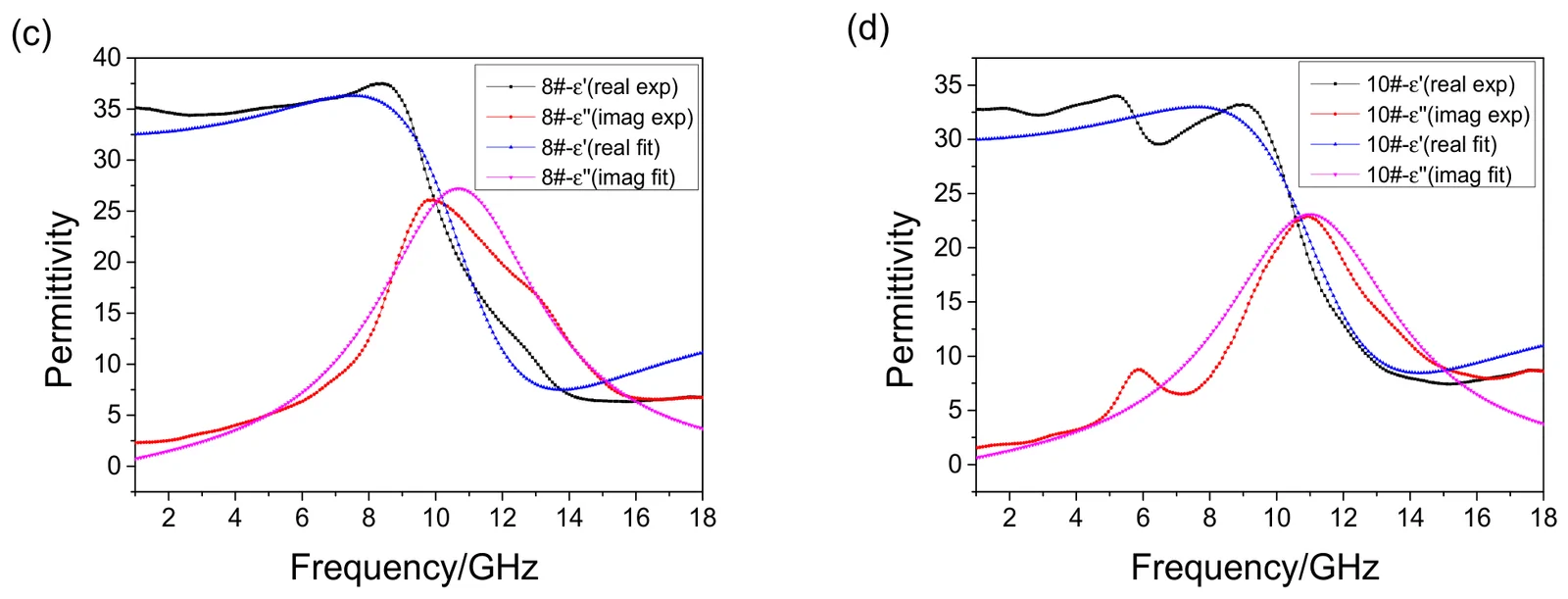
The fitting results of the Lorentz model of typical samples (**a**) 1# (**b**) 6# (**c**) 8# (**d**) 10#.

We employed the least squares method to fit the electromagnetic parameters of samples 1# through 12# according to the formula for the complex dielectric constant of the model. The typical fitting results are presented in Figure 8:

The fitting results demonstrate that the fitted data of samples 1#, 6#, and 8# are in good agreement with the actual measurements. This suggests that under low power and short sputtering time conditions, the electromagnetic parameters of the coated laminate follow the Lorentz model, which characterizes resonant absorption behavior, with resonance frequencies primarily ranging from 11 GHz to 14 GHz. Based on the previous surface resistance analysis, it is evident that the coating on the fabric has not formed a conductive network. As a result, free electrons within the nano-scale nickel layer are confined to oscillating within limited spaces, leading to dielectric constant characteristics consistent with the Lorentz model. Meanwhile, the imaginary parts of the electromagnetic parameters for sample 10# exhibit a double-peak phenomenon, indicating the presence of two distinct Lorentz resonant mechanisms. In this case, the expression for the complex dielectric constant becomes [39]:

$$\varepsilon^{*}\left(\omega \right)=\varepsilon_{\infty }+\frac{\Delta \varepsilon_{1}\omega_{0,1}^{2}}{\omega_{0,1}^{2}-\omega^{2}+j\gamma_{1}\omega }+\frac{\Delta \varepsilon_{2}\omega_{0,2}^{2}}{\omega_{0,2}^{2}-\omega^{2}+j\gamma_{2}\omega }$$

**Figure 9:**
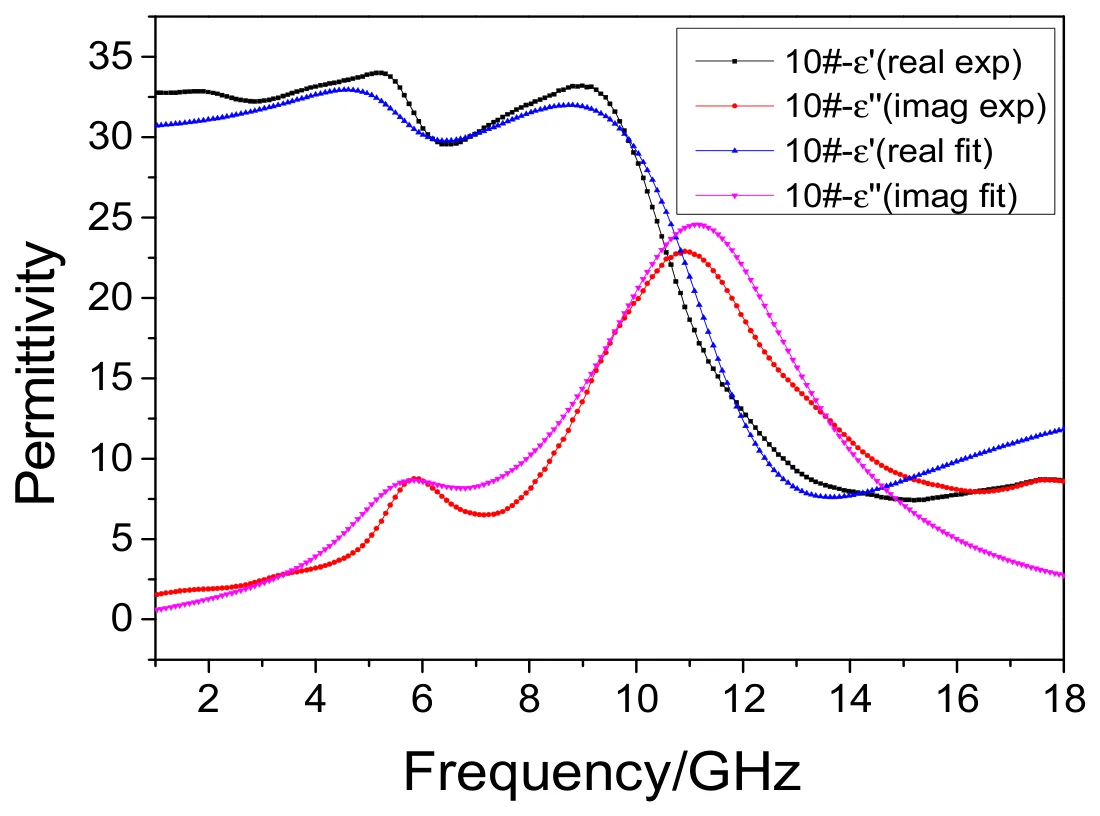
Fitting of the double Lorentz model for samples 10#.

Among these parameters, $\Delta \varepsilon_{1}$ denotes the change in the dielectric constant of the first Lorentz polarization, $\omega_{0,1}$ represents the resonance frequency of the first Lorentz polarization, and $\gamma_{1}$ is the damping coefficient of the first Lorentz polarization. $\Delta \varepsilon_{2}$ indicates the change in the dielectric constant of the second Lorentz polarization, $\omega_{0,2}$ is the resonance frequency of the second Lorentz polarization, and $\gamma_{2}$ is the damping coefficient of the second Lorentz polarization. $\varepsilon_{\infty }$ stands for the high-frequency dielectric constant. The fitting results of its electromagnetic parameters are presented in Figure 9:

The results presented in the figure demonstrate that the double Lorentz model provides a more accurate description of the loss mechanisms in samples 10#. From the observed variation patterns, it is evident that the appearance of double peaks is primarily correlated with sputtering time. Regardless of the sputtering power, double peaks emerge as long as the sputtering time is sufficiently long. Furthermore, with increasing sputtering time, the resonant frequency of the low-frequency peak shifts toward lower frequencies. Combining these findings with previous analysis of electron microscopy results, it can be concluded that the nano-nickel films are not uniformly distributed across the fibers. Fibers directly facing the sputtering target accumulate the thickest coatings, whereas the outer fibers shield the inner ones, allowing only a limited number of nickel atoms to penetrate and deposit on the inner fibers. Consequently, the coating thickness on the inner fibers is significantly reduced. Therefore, as sputtering time increases, the difference in coating thickness between inner and outer fibers becomes more pronounced, leading to the emergence of the double Lorentz resonant peaks.

### 3.5. Analysis of the Absorption Performance

According to the transmission line theory, the electromagnetic wave absorption performance of laminated plates with different thicknesses can be calculated based on the electromagnetic parameters of the materials. The calculation formula is as follows [40]:

$$R=-20\log_{10}\left|\frac{Z_{in}-Z_{0}}{Z_{in}+Z_{0}}\right|$$

Here:

$$Z_{in}=\eta tanh\left(\gamma d\right)$$

$$Z_{0}=\sqrt{\frac{\mu_{0}}{\varepsilon_{0}}}$$

**Figure 10:**
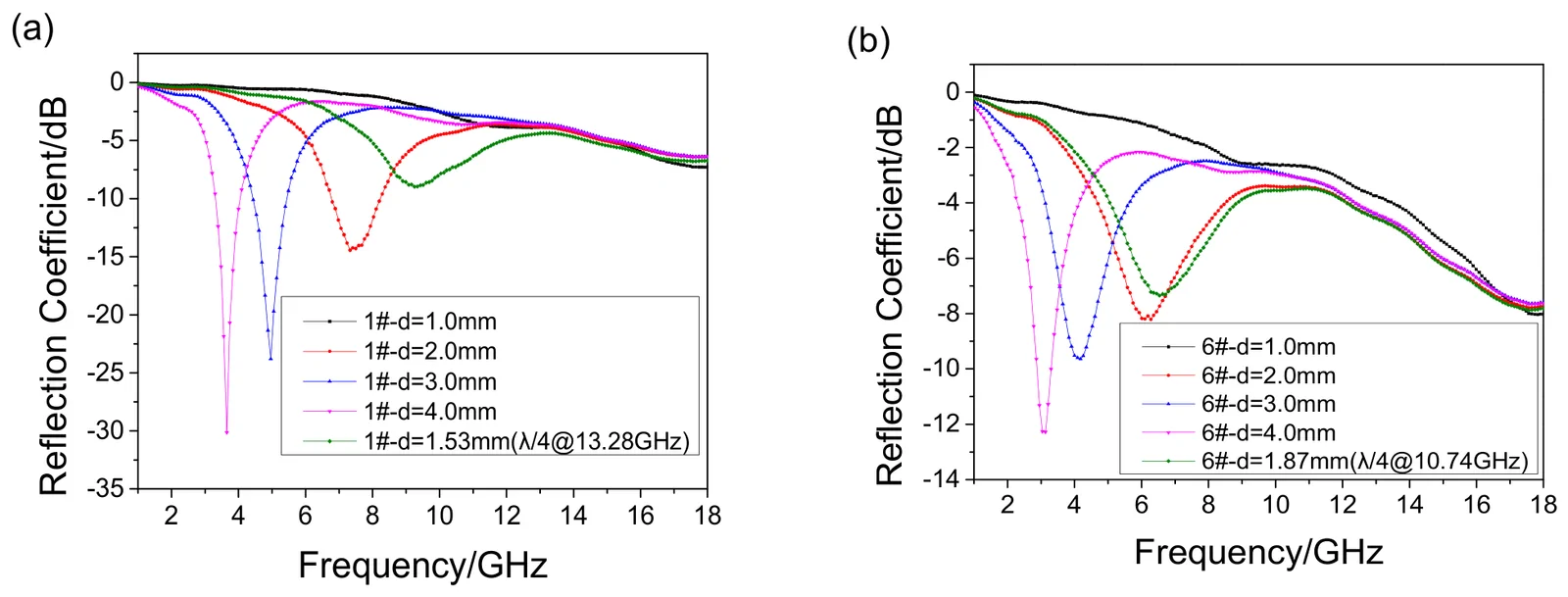

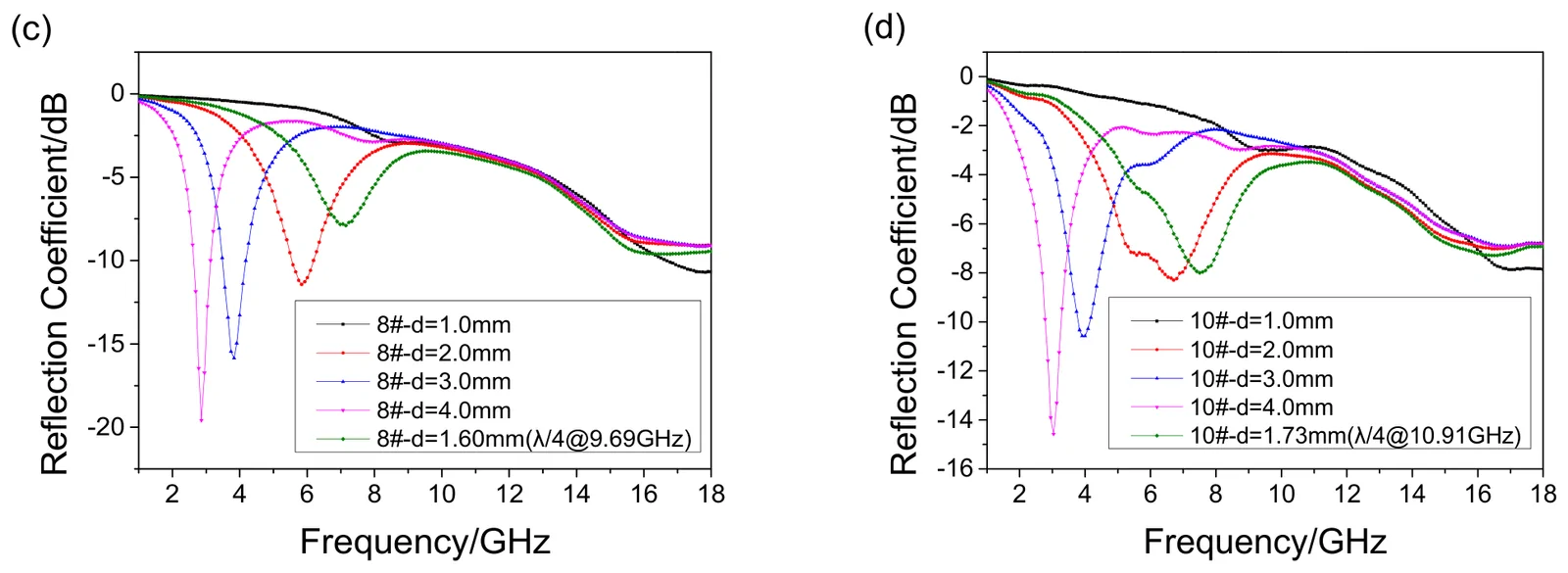
Variation trend of absorption performance of typical samples with different laminate thickness (**a**) 1# (**b**) 6# (**c**) 8# (**d**) 10#.

Based on the above formula, the reflectivity of the single-layer absorbing material for samples 1#, 6#, 8#, and 10# was calculated and is shown in Figure 10:

Figure 10 presents the simulation results of the reflectivity of the single-layer laminates of samples 1#, 6#, 8#, and 10# as a function of the thickness of the absorbing material. From the reflectivity data, it can be observed that due to the consistent type of frequency dispersion of the material’s electromagnetic parameters, the single-layer absorption performance of samples 1#, 6#, 8#, and 10# varies with thickness in a largely consistent manner. Under the same sputtering power, as the sputtering time increases, the absorption peak position at the same thickness shifts slightly towards lower frequencies. Under the same sputtering time, as the sputtering power increases, the absorption peak position at the same thickness shifts more notably towards lower frequencies; yet, the depth of the absorption peak becomes shallower. This suggests that as the sputtering power rises, the impedance-matching performance deteriorates. Meanwhile, for samples 10# featuring distinct dual resonant frequencies, when the thickness is 2 mm, dual absorption peaks emerge, and the absorption bandwidth broadens. We also computed the quarter-wavelength at the maximum loss resonant frequency point for each sample and utilized this as the material thickness to calculate the reflectivity, as depicted by the red solid line. The calculation results demonstrate that there is no substantial improvement in the absorption performance. This implies that a single-layer material cannot achieve strong absorption at the resonant point, and additional electrical structure design is necessary to attain better absorption performance.

**Figure 11:**
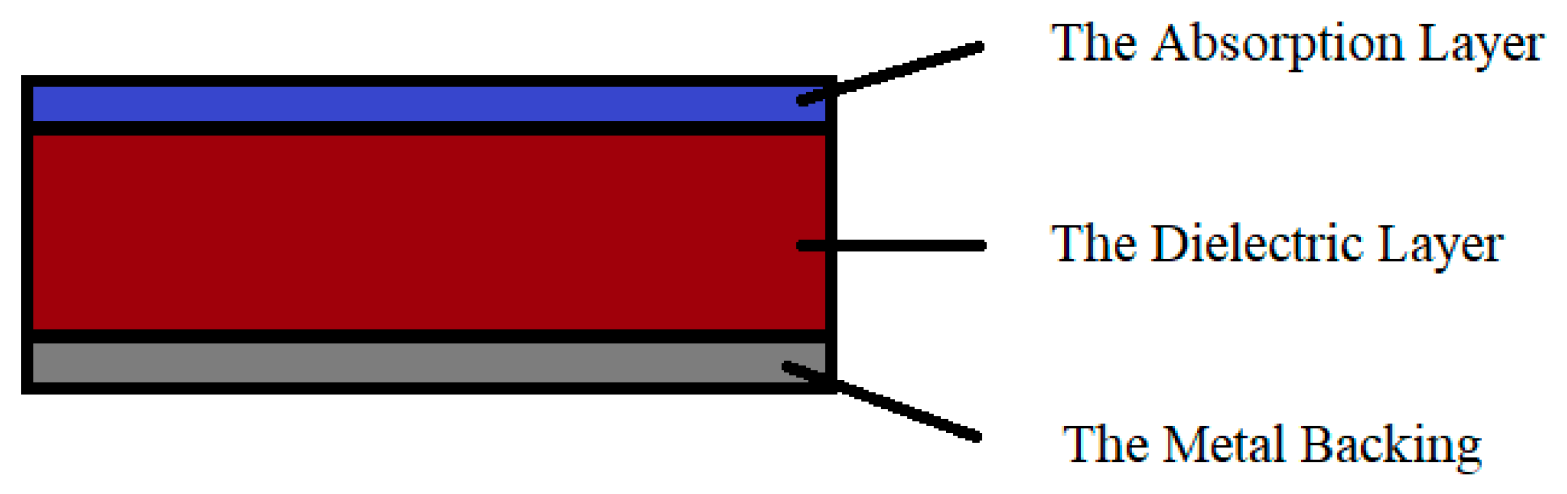
The structure of the absorber.

**Figure 12:**
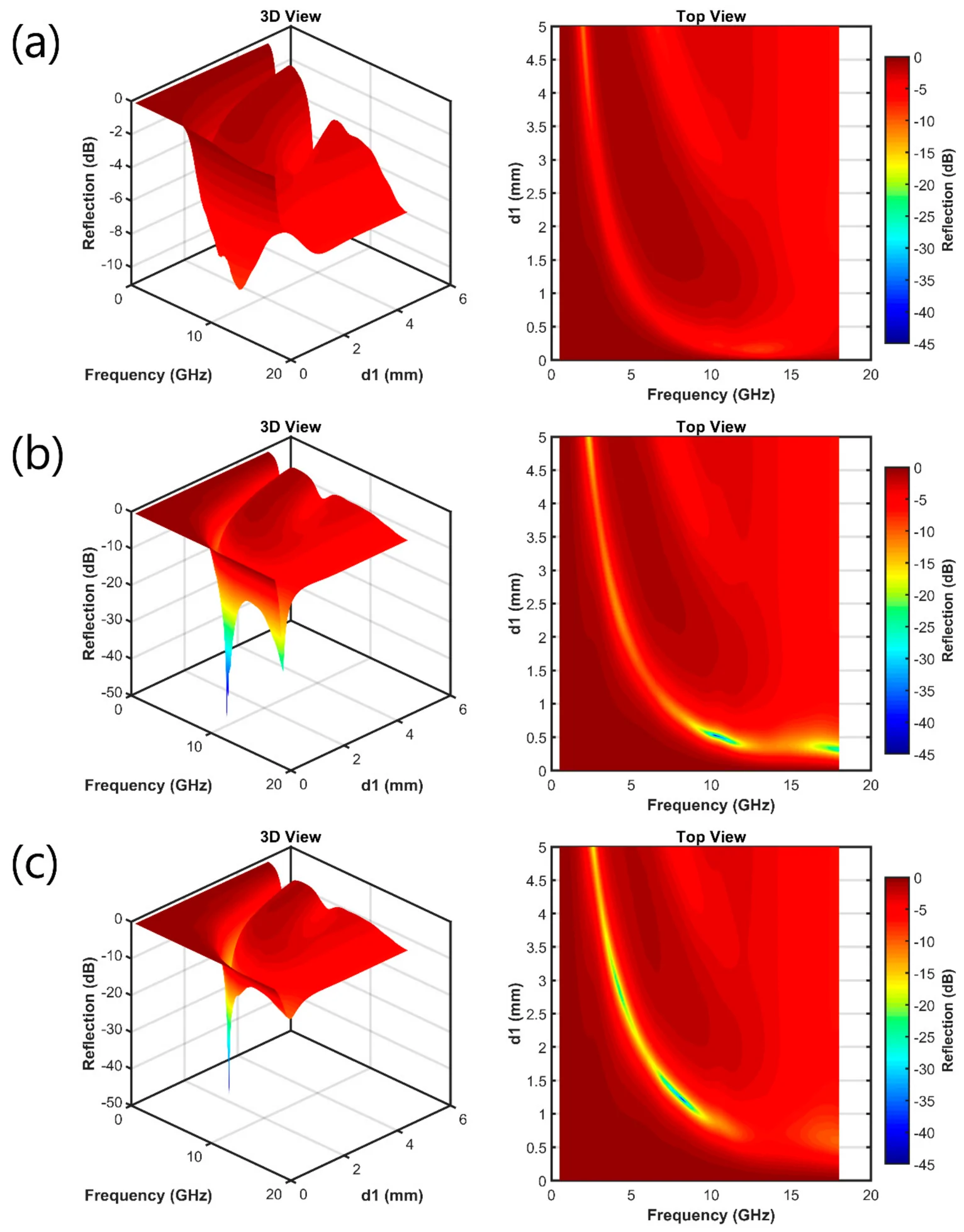
Simulation calculation results of Sample 1# and the matching layer were determined to be (**a**) 2.75 mm, (**b**) 1.38 mm, and (**c**) 0.69 mm.

To further improve the microwave absorption performance of the coated laminated plate, we optimized the structure of the absorber by incorporating a dielectric matching layer beneath the absorption layer based on the design concept of the Salisbury screen [41]. The dielectric matching layer is formed by laying up and curing a prepreg made from EW100A glass fabric and ME301 epoxy resin, with an areal density of 170 g/m^2^ and a cross-ply lay-up, and the curing process is identical to that used in this research project. Using MATLAB (version 2018R), we implemented a simulation program based on the transfer matrix method to compute the reflectivity of multilayer structures. Simulation calculations were then performed. The electromagnetic parameters of the dielectric matching layer are as follows: the real part of the dielectric constant is 4.2, the loss tangent is 0.021, the magnetic permeability is 1, and the magnetic loss is negligible. The proposed design model is illustrated in Figure 11:

The thickness of the dielectric matching layer (d2) was set to one quarter, one eighth, and one sixteenth of the resonant wavelength, respectively. Taking sample 1# as an example, which has a resonant frequency of 13.28 GHz, the corresponding thicknesses of the dielectric matching layer were determined to be 2.75 mm, 1.38 mm, and 0.69 mm, respectively. The thickness of the absorbing layer (d1) was also optimized, and the simulation results are presented in Figure 12:

Figure 12 illustrates the variation in absorption performance of Sample 1# with respect to the thickness of the absorbing layer under different thicknesses of the dielectric matching layer. It can be observed that the quarter-wavelength-matching layer does not contribute to the broadening of the material’s absorption bandwidth. When the absorbing layer thickness is 0.2 mm, the resonant peak appears at approximately 13 GHz, which aligns well with the resonant frequency predicted by the Lorentz model. However, when the matching layer thickness is reduced to one eighth of the wavelength, a significant expansion of the absorption peak is observed. At an absorbing layer thickness of around 0.3 mm, dual absorption peaks emerge in both the X-band and Ku-band, achieving absorption levels exceeding −10 dB across the 10~18 GHz frequency range. Further reducing the matching layer thickness to one sixteenth of the wavelength results in a shallower high-frequency absorption peak, a narrower absorption band, and an overall degradation in performance.

**Figure 13:**
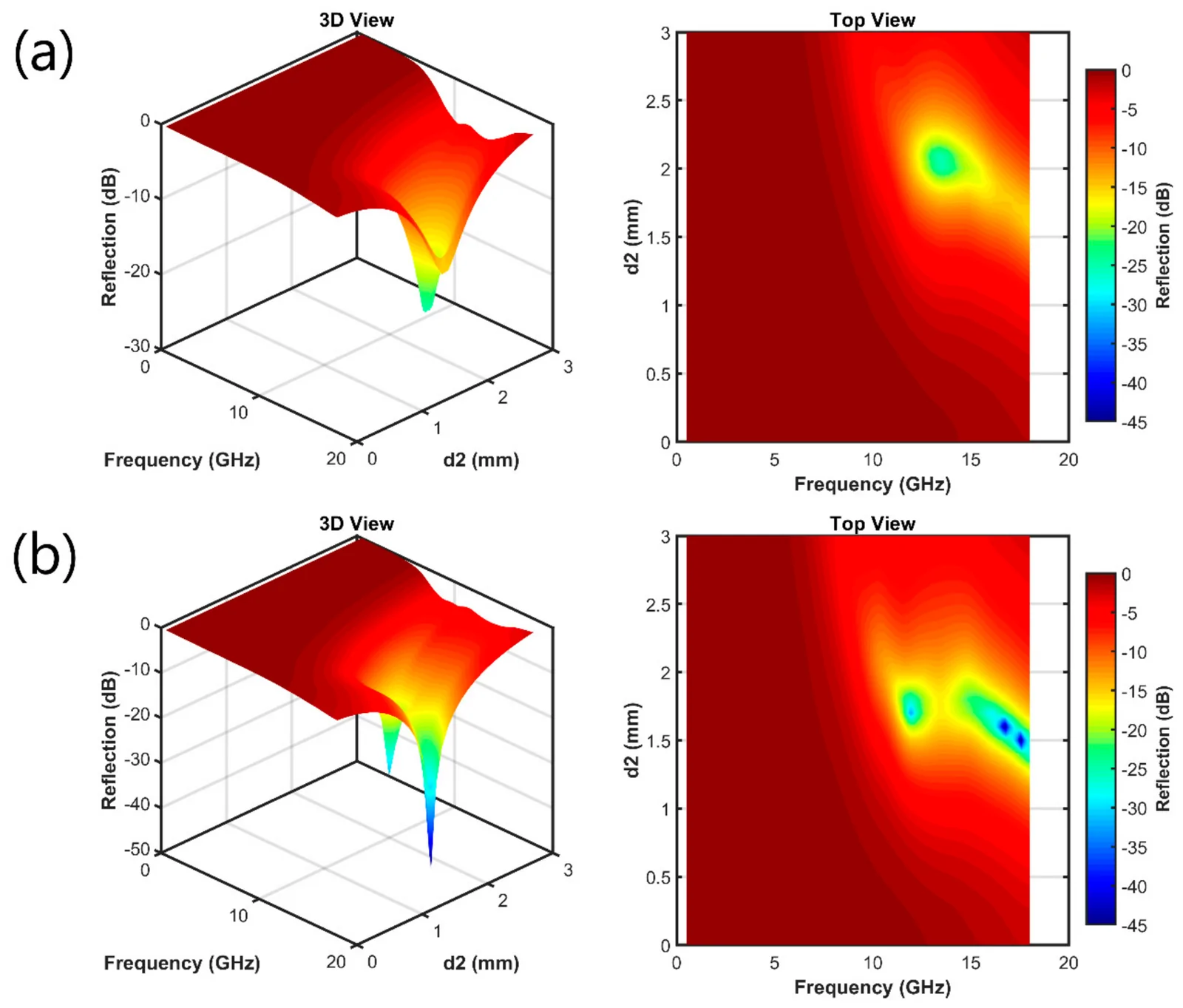

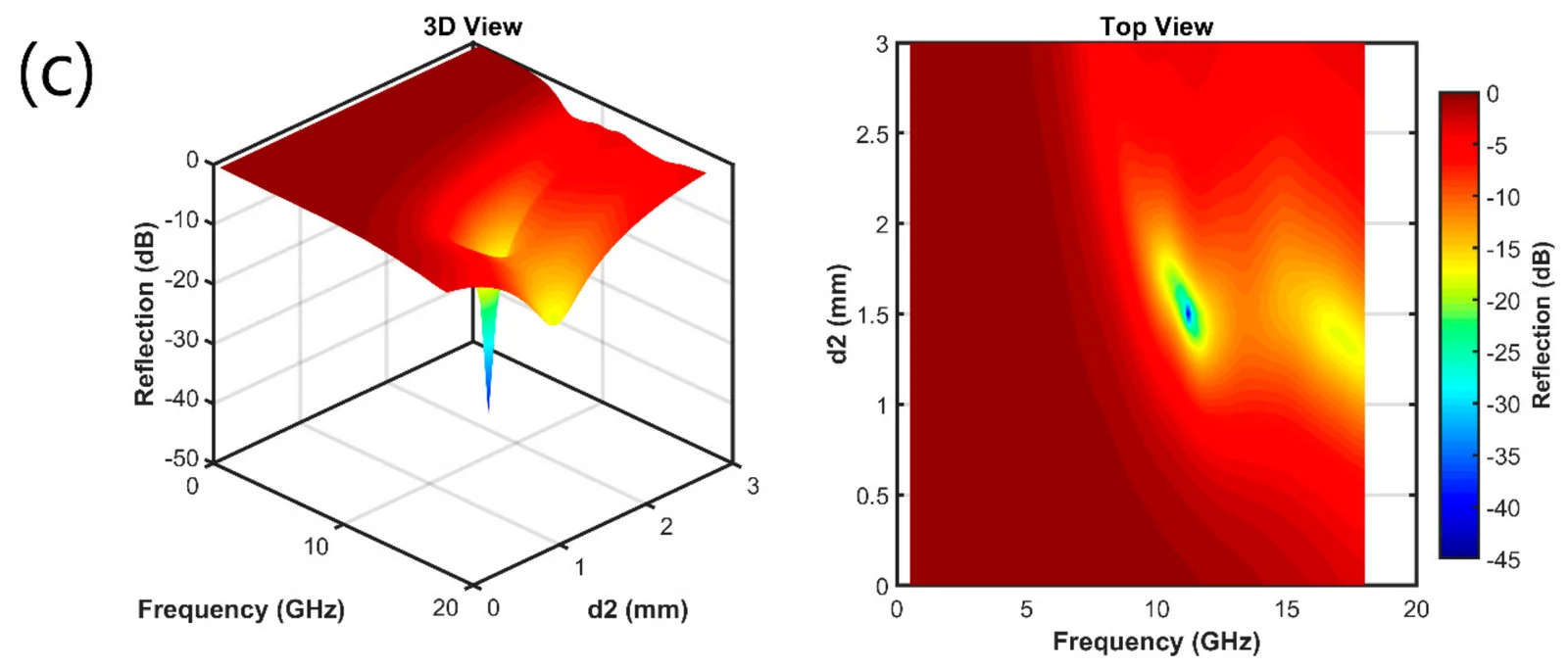
Optimization results of matching layer with different absorbing layer (**a**) 0.2 mm, (**b**) 0.3 mm, (**c**) 0.4 mm.

From the above results, it is evident that for Sample 1#, an optimal matching layer thickness exists when the absorbing layer thickness ranges from 0.2 mm to 0.4 mm. Accordingly, we selected absorbing layer thicknesses of 0.2 mm, 0.3 mm, and 0.4 mm, and further optimized the corresponding matching layer thicknesses. The simulation results are shown in Figure 13:

When the absorbing layer thickness is 0.2 mm, the optimal matching layer thickness is 2 mm, enabling deep absorption in the Ku-band. When the absorbing layer thickness increases to 0.3 mm, the optimal matching layer thickness decreases to approximately 1.7 mm, achieving strong absorption across the 10–18 GHz frequency range. Further increasing the absorbing layer thickness to 0.4 mm results in an optimal matching layer thickness of 1.5 mm, which also enables effective absorption within the 10–18 GHz range.

**Figure 14:**
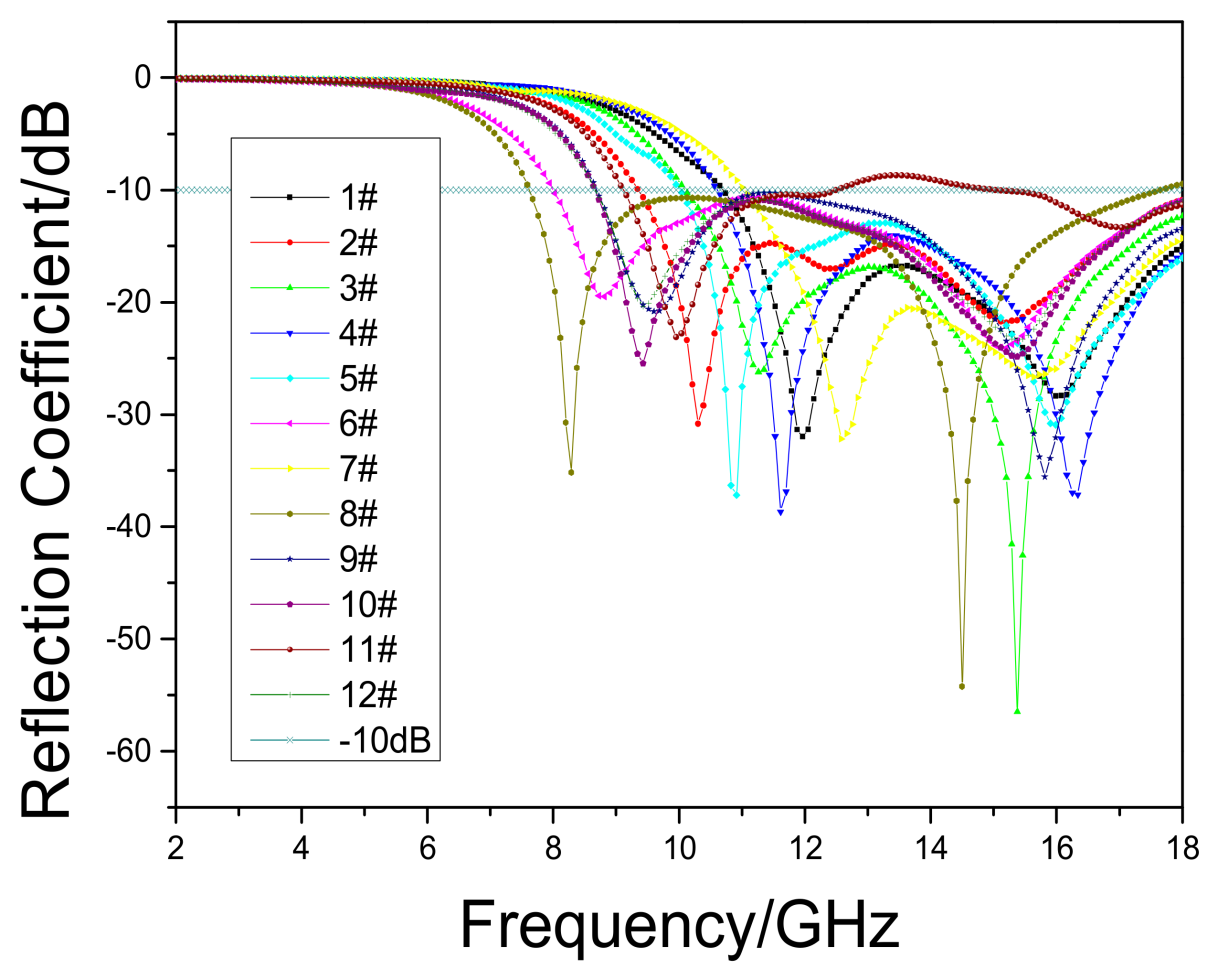
The Optimized Results.

Based on the above research process, the structure design was carried out for all samples. When the total thickness was controlled within 2 mm ± 0.1 mm, the simulation curve of the reflectivity is shown in Figure 14:

Figure 14 shows that after the electrical structure design, all samples can basically achieve absorption of ≤−10 db within the range of 11 to 18 GHz. Among them, samples 6# and 8# can achieve absorption of ≤−10 db within a wider frequency band of 8 to 18 GHz.

### 3.6. Reflectivity Testing of Laminates

According to the above-mentioned absorber structure design, laminates of samples 6# and 8# were prepared, with a sample size of 300 mm × 300 mm.

**Figure 15:**
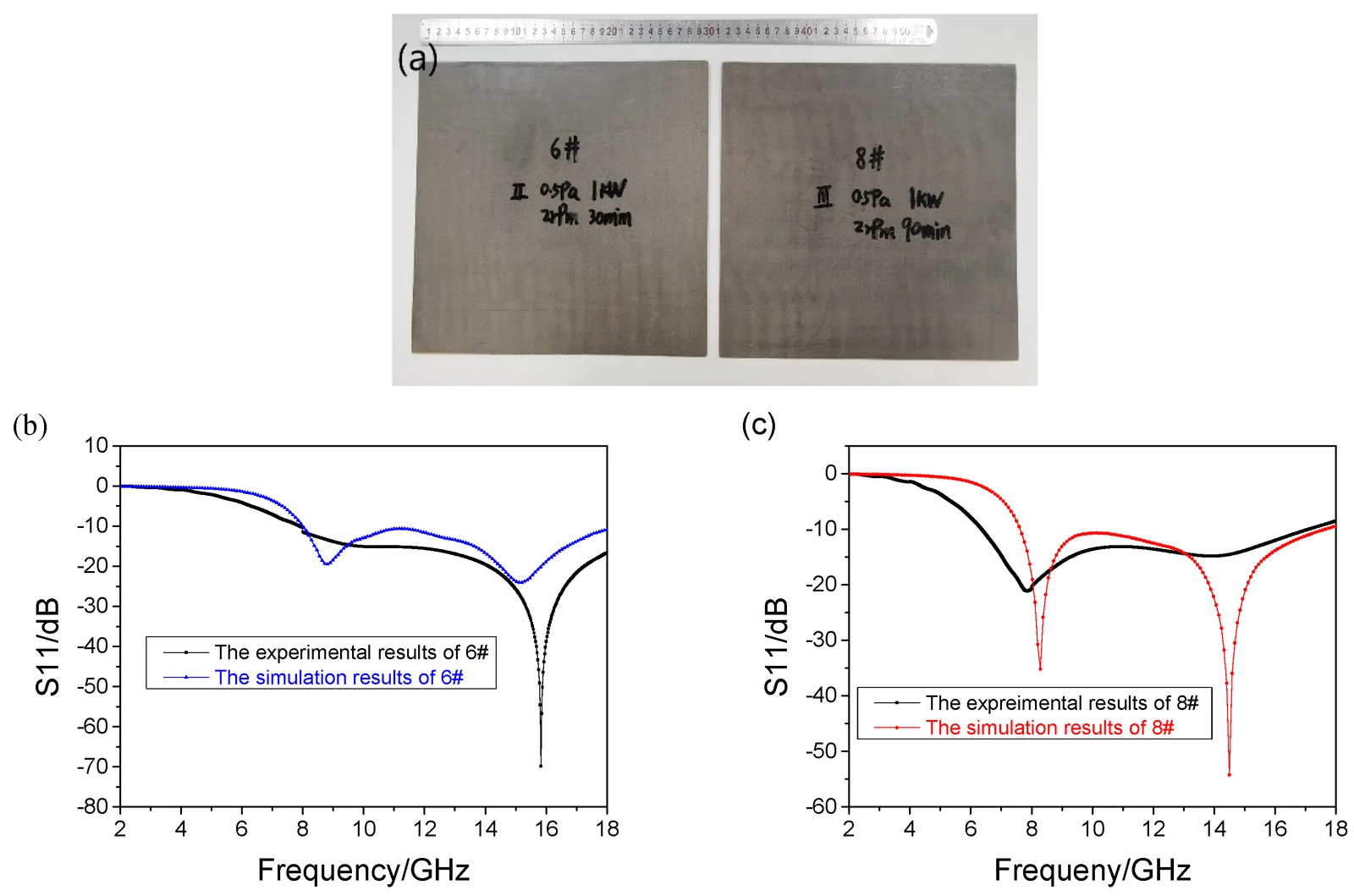
The bow method (**a**) The test sample and the comparison of the experimental results and simulation results of (**b**) 6# and (**c**) 8#.

As shown in Figure 15, the reflectivity curves of samples 6# and 8# exhibit dual absorption peaks, both achieving broadband absorption within the frequency range of 8–18 GHz. For sample 6#, the absorption peaks are located at 9.5 GHz and 16 GHz, showing a shift toward higher frequencies compared to the simulation results. In contrast, sample 8# exhibits absorption peaks at 7.5 GHz and 14 GHz, indicating a shift toward lower frequencies relative to the simulated values. These shifts can be attributed to the Lorentzian relaxation absorption mechanism of the material. As observed from the simulation results illustrated in Figure 12 and Figure 13, materials exhibiting the Lorentzian relaxation behavior are highly sensitive to the thicknesses of both the absorption layer and the matching layer. Variations in these thicknesses result in corresponding changes in the positions and depths of the absorption peaks. Based on the observed trends, it is evident that the actual thicknesses of the absorption and matching layers in sample 6# are smaller than those used in the simulations, whereas those in sample 8# are greater, thereby causing the respective peak shifts.

**Figure 16:**
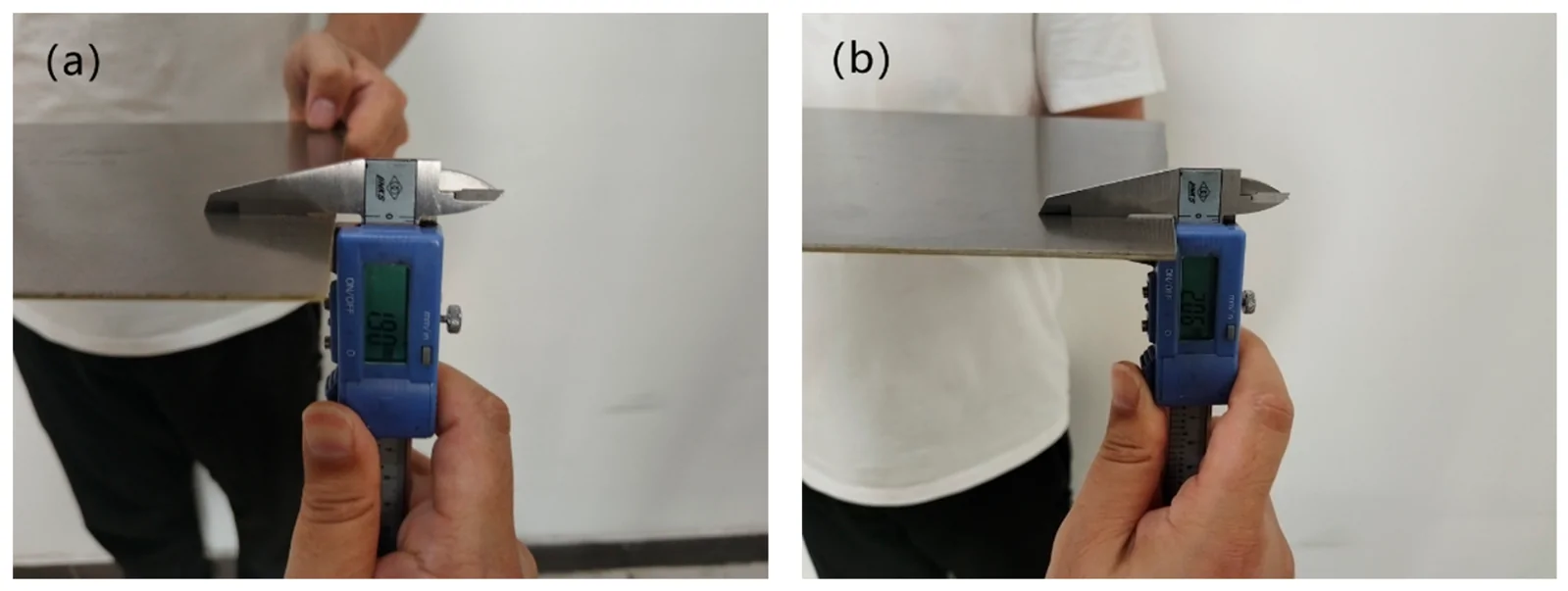
The thickness of different samples (**a**) 6# (**b**) 8#.

**Figure 17:**
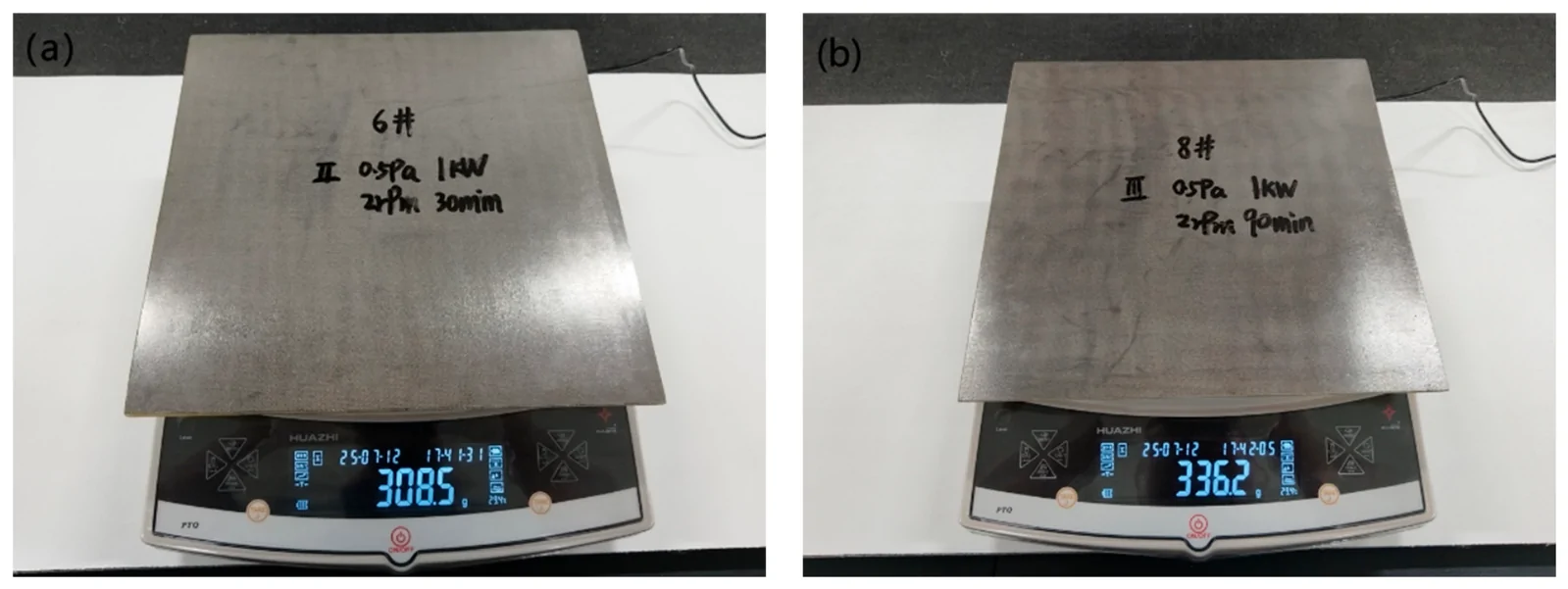
The weight of different samples (**a**) 6# (**b**) 8#.

According to the test results presented in Figure 16 and Figure 17, Sample 6 has a weight of 308.5 g and a thickness of 1.9 mm, whereas Sample 8 weighs 336.2 g and has a thickness of 2.06 mm. By incorporating the sample dimensions, the bulk densities of both samples were calculated. The resulting bulk density for Sample 6 is 1.8 g/cm^3^, and for Sample 8 it is 1.81 g/cm^3^, indicating a notable reduction compared to the bulk density of conventional structural absorbing composites that incorporate magnetic absorbent.

Furthermore, a systematic comparative analysis was conducted on representative studies of metallic nickel-based microwave absorbing materials reported in recent years, with particular emphasis on four critical performance parameters: the synthesis method, sample thickness, maximum reflection loss (RL), and effective absorption bandwidth (EAB), defined as the frequency range over which RL ≤ −10 dB. A summary of these parameters is presented in Table 6.

Based on the comparative data summarized in the table, the present work demonstrates the most outstanding overall microwave absorption performance among all the referenced studies. The Ni-coated glass fiber fabric, fabricated by DC magnetron sputtering, achieves a minimum reflection loss (RL_min) of −69.8 dB at a matching thickness of only 1.9 mm, which is significantly superior to the best reported values in the literature. In terms of effective absorption bandwidth (EAB), the present sample covers the entire 8–18 GHz frequency range with a bandwidth of 10 GHz, outperforming all the other absorbers, including the nichrome/quartz fabric (7.0 GHz, 8.5–15.5 GHz) and the Ni-N@C composite (5.21 GHz). Notably, this broadband performance is achieved with a thickness of 1.9 mm, whereas the only comparable bandwidth (12.5 GHz) reported elsewhere relies on a complex three-layer configuration with a thickness of 5 mm. Overall, this study not only sets a new benchmark for reflection loss and effective bandwidth in magnetron-sputtered metal/fabric composites but also presents a cost-effective and industrially viable strategy for designing high-performance, lightweight, wideband microwave absorbers.

## 4. Conclusions

In this paper, continuous fiber-reinforced nickel-coated microwave absorbing composites were successfully fabricated, and their microwave absorption performance was optimized. Single-layer materials exhibited limited performance, but by incorporating a dielectric matching layer, a composite structure with a total thickness of only 2 mm achieved significantly broadened absorption bandwidth. Specifically, samples 6# (1 kW, 30 min) and 8# (1 kW, 90 min) showed broadband absorption (≤−10 dB) within the 8–18 GHz range, with experimental data matching simulation results. By precisely controlling the coating process and electrical structure, magnetron-sputtered nickel-coated continuous glass fiber composites can realize excellent broadband absorption, offering a promising route for lightweight, high-performance absorbers. Future work will focus on further enhancing the coating’s magnetic properties to improve low-frequency absorption.

## Figures and Tables

**Table 1:** Different process parameters.

Sample Number	Sputtering Power (kW)	Sputtering Time (min)	Sample Number	Sputtering Power (kW)	Sputtering Time (min)
1#	0.5	10	7#	1	60
2#	0.5	30	8#	1	90
3#	0.5	60	9#	2	10
4#	0.5	90	10#	2	30
5#	1	10	11#	2	60
6#	1	30	12#	2	90

**Table 2:** Test equipment used in this project.

Equipment	Model
Scanning Electron Microscope	JEOL JSM-7500F (JEOL, Tokyo, Japan)
X-ray Diffractometer	Rigaku SmartLab 9 kW (Rigaku, Tokyo, Japan)
Vector Network Analyzer	Agilent N5244A (Agilent, Santa Clara, CA, USA)
Arch measurement system	-

**Table 3:** The thickness of the coating on each sample.

Sample Number	Thickness (nm)					Maximum Difference (nm)
	Position 1	Position 2	Position 3	Position 4	Position 5	
1#	40.26	49.58	39.47	95.4	50.18	55.93
2#	83.74	52.96	134.4	120.3	122.4	81.44
3#	260.5	228.9	173.4	256.1	265.9	92.5
4#	282.5	205.4	388.8	240.3	381.3	183.4
5#	124.8	72.57	184.6	72.57	63.16	121.44
6#	255.8	216.8	197.8	217.4	199.9	58
7#	489.7	309.6	367.3	519	400.1	209.4
8#	533.2	291.3	694.2	78.15	337.2	606.05
9#	93.58	173.3	129.5	172.3	159.7	79.72
10#	319.7	325.1	270	162.6	318.7	162.5
11#	499.6	904.2	425.8	727.9	857.9	478.4
12#	1356	1091	1382	426.1	619.9	955.9

**Table 4:** Positions of Diffraction Peaks and the Lattice parameters.

Sample Number	Diffraction Peak Angle and Intensity						Lattice Parameters (Å)
	(111)	Area%	(200)	Area%	(220)	Area%	
1#	44.281	100	-	-	-	-	3.5288
2#	44.396	100	-	-	-	-	3.5244
3#	44.414	100	51.868	16.2	76.403	5.0	3.5249
4#	44.375	100	51.774	19.9	76.325	19.6	3.5270
5#	44.432	100	51.613	5.4	-	-	3.5337
6#	44.409	100	51.783	16.3	76.452	11.8	3.5251
7#	44.356	100	51.716	20.7	76.343	17.0	3.529
8#	44.428	100	51.870	32.8	76.359	17.4	3.5114
9#	44.383	100	51.949	6.7	76.288	3.6	3.5244
10#	44.418	100	51.760	26.6	76.422	10.9	3.5271
11#	44.330	100	51.715	21.8	76.290	16.5	3.5311
12#	44.398	100	51.788	26.1	76.366	36.7	3.5274

**Table 5:** Test results of surface resistance of Ni-coated fiber fabric.

Sample Number	Resistance (Ω·m)	Sample Number	Resistance (Ω·m)
1#	3.738 × 10^8^	7#	3.054 × 10^8^
2#	2.109 × 10^8^	8#	5.056 × 10^8^
3#	4.114 × 10^8^	9#	5.842 × 10^8^
4#	3.423 × 10^8^	10#	6.898 × 10^8^
5#	3.809 × 10^8^	11#	4.664 × 10^8^
6#	2.001 × 10^8^	12#	3.090 × 10^8^

**Table 6:** The comparative analysis of some previous reports.

Reference	Fabrication Method	Thickness (mm)	RL_min (dB)	RL_min (dB)
FeNi/C nanofibers [42] (Lv et al., 2018)	Electrospinning + heat treatment (700 °C carbonization)	2.7 (for RL_min) 1.8 (for EAB)	−24.8 (at 9.4 GHz)	4.4 GHz (12.8–17.2 GHz)
Nichrome/quartz fabric [31] (Wang et al., 2020)	DC magnetron sputtering (Ni–Cr 80/20)	~3 (total)	−53 (at 11.48 GHz)	7.0 GHz (8.5–15.5 GHz)
Ni–N@C composite [43] (Li et al., 2020)	Hydrothermal synthesis + polypyrrole coating + carbonization (800 °C)	3.0 (for RL_min) 2.0 (for EAB)	−32.31 (at 9.06 GHz)	5.21 GHz (12.79–18.0 GHz)
MXene/Ni/aluminosilicate glass [44] (Luo et al., 2021)	Zeolite ion-exchange + in situ reduction + spark plasma sintering (700 °C)	2.35 (for RL_min) <4.5 (for EAB)	−59.5 (at 10.8 GHz)	4.1 GHz (Ku-band)
F–CNTs@Ni/epoxy [27] (Liu et al., 2022)	CVD growth of CNTs + electroless Ni plating + resin impregnation	Multilayer: 3.5 + 2.6 + 0.875 (for RL_min) 2.5 + 1.4 + 1.1 (for EAB)	−60	12.5 GHz (5.5–18 GHz) 5.6 GHz (3.9–12.3 GHz)
Ni/C nanofibers [45] (Zhang et al., 2024)	Electrospinning + carbonization (600 °C, H_2_/Ar)	3.0	−30.6	−30.6
This work	DC magnetron sputtering (Ni)	1.9 mm	−69.8	10 GHz (8~18 GHz)

## Data Availability

The raw data supporting the conclusions of this article will be made available by the authors on request.
